# Multi-scale computational modeling of magnetoelectric nanoparticle-dispersed conductive nanofibers for peripheral nerve modulation

**DOI:** 10.3389/fbioe.2026.1951247

**Published:** 2026-10-13

**Authors:** Valentina Galletta, Serena Fiocchi, Claudio Verardo, Luigi Carbone, Loretta Laureana del Mercato, Simone Romeni, Emma Chiaramello

**Affiliations:** 1 Department of Electronics, Information and Bioengineering (DEIB), Politecnico di Milano, Milan, Italy; 2 Institute of Electronics, Computer and Telecommunication Engineering (IEIIT), National Research Council (CNR), Milan, Italy; 3 The BioRobotics institute, Health Science Interdisciplinary Research Center, and Department of Excellence Robotics and AI, Scuola Superiore Sant’anna, Pisa, Italy; 4 Institute of Nanotechnology, National Research Council (CNR NANOTEC), Lecce, Italy; 5 Istituto Italiano di Tecnologia, Center for Biomolecular Nanotechnologies, Arnesano (LE), Italy; 6 Tecnomed Puglia - Technopole for Precision Medicine (Biotech Lecce Hub), Lecce, Italy; 7 Modular Implantable Neurotechnologies (MINE) Laboratory, Università Vita Salute San Raffaele and Scuola Superiore Sant’Anna, Milan, Italy

**Keywords:** computational modelling, core-shell magnetoelectric nanoparticles, hybrid biointerfaces, noninvasive stimulation, wireless peripheral nervous stimulation

## Abstract

**Introduction:**

The electrical stimulation of peripheral nerves has shown the potential to restore impaired functions across a wide range of clinical scenarios. In most cases, though, the lack of selectivity of existing stimulation interfaces limits their applicability and can in some cases produce severe adverse effects. In addition, traditional stimulation requires physical connectors to a pulse generator that can add further biological and mechanical stress already linked to interfacing with physiological structures. This study focuses on the use of magnetoelectric nanoparticles (MENPs) embedded within conductive polymeric nanofibers as new wireless, highly selective, and minimally invasive interfaces for modulating peripheral nerves activity.

**Methods:**

A step-by-step detailed characterization of the physical variables involved in this interaction, spanning from the nanometric single MENPs to micrometric multi-nanofibers structure with dispersed MENPs (poly-ME devices) and ending with the millimetric peripheral nerve, was proposed. MENPs concentration and distribution, device geometry, orientation, and size were systematically varied, and their effects on nerve fiber recruitment were quantified across myelinated and unmyelinated fiber types at different distances from the stimulating source.

**Results and discussion:**

Our results evidenced how higher MENPs concentrations, higher stimulating source size and lower distance enhance the modulation capability of the poly-ME devices, with differences in stimulation capability between myelinated and unmyelinated nerve fibers. The solution of embedding MENPs in conductive polymer nanofibers enables higher MENPs loading than direct tissue injection, improves positional stability, and preserves biocompatibility, thereby offering a potential alternative for the engineering of MENPs as neuromodulation interfaces.

## 1 Introduction

Electrical stimulation is a well-established method used to introduce information into the nervous system, with the final aim of exerting external control on the biological system or restoring impaired functions (Grill et al., 2009; Benabid, 2003; Wagner et al., 2018; Peckham and Knutson, 2005; Birmingham et al., 2014). Increasing interest has been shown in peripheral nerve stimulation (PNS), since it allows reduced invasiveness with respect to targeting the central nervous system directly and can lead to increased selectivity by stimulating structures where functional segregation tends to be high. PNS has enabled the development of innovative therapeutic approaches ranging from the treatment of neuropathic pain (Slavin, 2008; Petrini et al., 2019) and the regulation of bodily (autonomic) functions (Birmingham et al., 2014; Cracchiolo et al., 2021), to the restoration of sensory feedback in amputees (Petrini et al., 2019; Raspopovic et al., 2014; Tan et al., 2014), and the reinstatement of coordinated muscle contractions in completely paralyzed patients (Micera et al., 2026).

This large number of PNS applications naturally comes with specific requirement in terms of necessary functional selectivity to limit side-effects (Grill et al., 2009; Navarro et al., 2005; Yildiz et al., 2020) and of surgical accessibility, potentially imposing the use of different neural interfaces, which establishes the direct contact with the nervous tissue (Zhang et al., 2020). For traditional neural interfaces, increased selectivity comes at the expense of increased invasiveness of the used electrodes, which is in turn directly related to both tissue damage and difficulty of implantation (Romeni et al., 2020). Even though flexible and stretchable electrodes made with polymeric substrates (Bettinger, 2018) and optical interfaces (Warden et al., 2014) can reduce the mechanical mismatch between the interface and its neural target, most neural interfaces are connected to pulse generators through wires whose presence increases the probability of tissue inflammation (Habibollahi et al., 2024; Koch et al., 2019).

Magnetoelectric nanomaterials are a new solution that could potentially overcome the current problems of mechanical mismatch and wired technologies. Their underlying physical principle is the magnetoelectric (ME) effect, i.e., the coupling between electric and magnetic fields, and it is distinguished between the direct and converse ME effects. Specifically, for the direct ME effect, the application of an external magnetic field generates a local electric field, and the magnitude of this coupling is defined by the direct ME coefficient, i.e., the ratio of the electric change in the material to the externally applied bias magnetic field (Eerenstein et al., 2006; Fiebig, 2005). The most common structures featuring the ME effect are generally two-phase multiferroic composite materials consisting of magnetostrictive and piezoelectric phases: different structures exhibit different ME coefficients, which strongly depend on both physical characteristics, such as material composition, geometry, and size, as well as fabrication processes, including mechanical mismatches between the two phases (Lawes and Srinivasan, 2011).

Among the various structures, the two-phase core-shell magnetoelectric nanoparticles (MENPs) have attracted particular attention for biomedical applications. MENPs based on cobalt ferrite (CoFe_2_O_4_, CFO) cores surrounded by a shell of barium titanate (BaTiO_3_, BTO) represent a model system that has proven biocompatibility and enhanced ME performance (Apu et al., 2022). To exploit their direct ME effect, an external bias magnetic field is applied to deform the magnetostrictive core. This deformation is then transferred to the piezoelectric shell via interphase mechanical coupling and at last, the piezoelectric properties of the shell transduce the mechanical change into a polarization change (Eerenstein et al., 2006; Fiebig, 2005) which could in turn affect the externally surrounding biological tissue. Overall, the combination of the ME effect and nanometric scale of MENPs indicates their potential as wireless and minimally invasive next-generation tissue interfaces, thereby suggesting the possibility of developing innovative electrodes that could enhance the performance of future bionic systems.

Some *in vitro* and *in vivo* experiments have demonstrated the potential of MENPs to stimulate neuronal activity in the central nervous system (CNS) (Kozielski et al., 2021; Nguyen et al., 2021; Zhang et al., 2022), but proper quantification of these interactions is difficult to achieve within these approaches. This highlights the importance of computational studies. By modeling the stimulation of MENPs within the target tissue, it is possible to provide a detailed characterization of the phenomenon, which can help both to understand the underlying physical mechanisms and to move toward a more optimized setup. Some computational studies have enabled quantification of the electrical effects of MENPs within the CNS (Chiaramello et al., 2022; Fiocchi et al., 2022a). However, few studies can be found on the application of this innovative technology as a stimulation tool for the peripheral nervous system.

An initial study (Galletta et al., 2024) explored the potential use of MENPs as tools for the PNS by modeling, as a first approximation, the effect of multiple MENPs agglomerated in clusters. A following study (Galletta et al., 2025) characterized the stimulating effect of multiple MENPs in a scenario with increased detail, by assuming MENPs distributed within a small volume of nerve tissue and quantifying the effect of varying nanoparticle concentrations and stochastic distributions. Despite the encouraging results, the effective delivery and distribution of MENPs to the target tissue remains an issue that requires further attention. A possible solution currently under investigation is the engineering of polymeric hybrid architectures, i.e., polymeric structures embedding nanoparticles. Structurally supporting the distribution of MENPs could ensure a more uniform distribution of nanoparticles in the targeted tissue and a better control over their insertion in the target tissue, which in turn can ensure a more uniform and enhanced control over the electrical stimulation that is delivered. In addition, the use of a polymeric support could stabilize the positioning of nanoparticles at the desired target location without loss of biocompatibility while ensuring portability and compliancy of the device, given the reduced mismatch of mechanical properties with the target tissue, as demonstrated by its use in enveloping conventional electrodes (Wurth et al., 2017). To date, the literature has documented the use of various natural and synthetic polymers to disperse nanoparticles, presenting a range of architectures such as random and aligned electrospun fibers or thin films (Tamo et al., 2024). Among the materials under investigation, conductive polymers are attractive due to their low electrochemical impedance, high capacities, and high biocompatibility (Jeong et al., 2015). A variety of applications are being explored for hybrid scaffolds, including tissue repair and regeneration (Zaszczyńska et al., 2024; Ferson et al., 2021), and controlled drug delivery in wound dressings (Baykara et al., 2023). However, there are limited studies focusing on the application of MENPs dispersed in polymeric structures and targeting the neuromodulation. An *in silico* study (Suarato et al., 2025) assessed the stimulation exerted on nerve fibers delivered by a 3D polymeric structure enclosing MENPs while characterizing the use of different polymers, and an *in vitro* study (Choe et al., 2024) evaluated the effectiveness of neuromodulation performed by MENPs dispersed within flexible and biodegradable nanofibers to enhance neuronal differentiation of neuron-like PC12 cells.

In this context, this study aims to provide further insight into the neuromodulation exerted on nerve fibers when MENPs are dispersed in polymeric structures. Specifically, MENPs were assumed dispersed within conductive polymeric electrospun nanofibers. Given its demonstrated biocompatibility, compliance and high electrical conductivity (Punjabi et al., 2015), the conductive polypyrrole (PPy) was considered as polymeric matrix, and multiple nanofibers were assumed to be aligned into a multi-nanofiber structure, referred to as poly-ME device. Computational methods were used to provide a quantification of the neuromodulation exerted on nerve fibers by leveraging on a comprehensive multi-step computational pipeline. This approach allowed, for the first time, the characterization of all the physical variables involved in the interaction, ranging from the nanometric scale of nanoparticles, moving to the micrometric scale of the poly-ME devices and ending with the millimetric scale of peripheral fascicles and nerves.

## 2 Materials and methods

### 2.1 Characterization of the direct ME effect of a single MENP

To quantify the electrical polarization on the surface of nanoparticles, the direct ME effect of a single MENP dispersed within a conductive polymeric medium was characterized (Figure 1A). The MENP exhibited a spherical shape with a core-shell structure, wherein a CFO was coated by a BTO shell. The total diameter was equal to 100 nm, with a shell thickness of 5 nm, resulting within the range of experimental studies (Betal et al., 2016; Pardo and Khizroev, 2022). All dielectric properties are listed in Supplementary Table S1. The conductive polymer in which the nanostructure was inserted was polypyrrole (PPy) with a conductivity of 1000 S m^-1^, within the range reported in the literature (Karagiorgis et al., 2025). An external DC magnetic field was applied to activate the direct ME effect of the MENP. To assess the electrical polarization on the MENP surface at its maximum amplitude (i.e., when the externally applied magnetic field is above magnetic saturation), the amplitude of the DC magnetic field was varied from 0 kOe to 10 kOe, as in (Kozielski et al., 2021; Kaushik et al., 2016).

**FIGURE 1:**
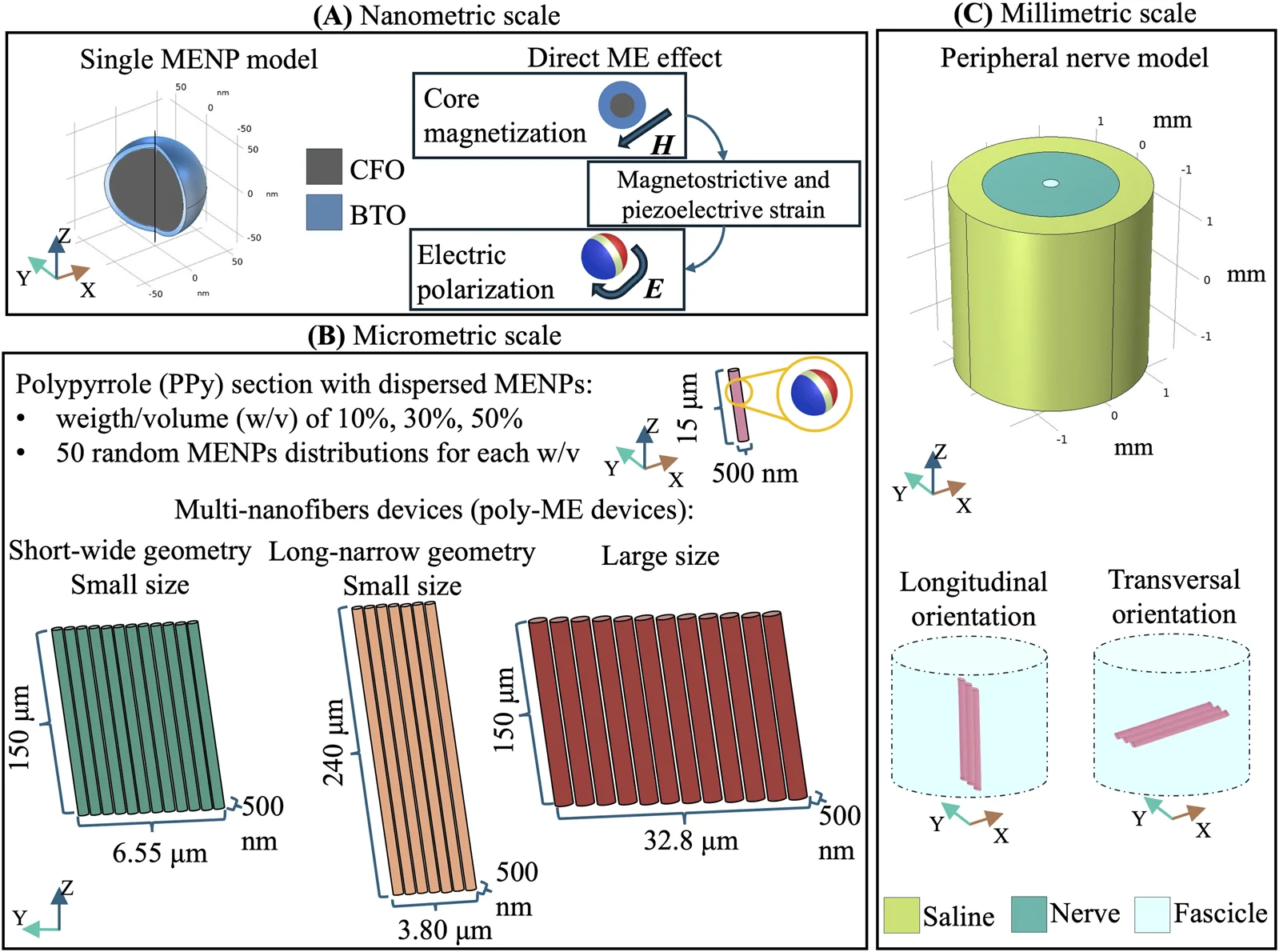
Multi-step computational modeling: **(A)** nanometric scale: geometric model of a single core-shell spherical MENP on the left, and the direct magnetoelectric effect on the right; **(B)** micrometric scale: on the top, visualization of a polypyrrole section with dispersed MENPs at varying concentration and random distribution, and at the bottom, multi-nanofibers polymeric magnetoelectric (poly-ME) devices with varying geometry (short-wide and long-narrow) and different size (small size and large size corresponding to a surface area of one-fifth and equal to a TIME electrode’s active site, respectively); **(C)** millimetric scale: on the top, visualization of the peripheral nerve geometry, with the fascicle of fibers, the nerve and the saline bath, and at the bottom, zoom to the interaction between the poly-ME device and the fascicle of fibers, illustrating the two different orientations analyzed (longitudinal and transversal).

The modelling was performed via a finite element method-based simulation tool, i.e., COMSOL Multiphysics 6.3 (COMSOL, 2024), following the methodology adopted in (Marrella et al., 2023; Bonato et al., 2025; Fiocchi et al., 2022b). The electrostatic, solid mechanics and magnetic field modules were used with the magnetostriction and piezoelectric effect multiphysics couplings (which were fully coupled) to evaluate the response of the MENP. A magnetic field boundary condition was applied to all the faces of the domain to simulate a uniform DC magnetic field, and the stationary study was then solved. For more details, please refer to the Supplementary Material.

### 2.2 Quantification of the electrical effect of multiple MENPs

The effect of multiple MENPs with random distribution and variable concentration inserted within a nanofiber section of PPy was then analyzed. Following the approach of (Fiocchi et al., 2022b), MENPs were approximated as dipoles aligned along the same direction, namely the one of the externally applied magnetic field which was used to activate their direct ME effect, assumed to be directed perpendicular to the height of the nanofiber section. The temporal behavior of activation of the MENPs was assumed to be sinusoidal with proper frequency to deal with the low-frequency dynamics of nerve fibers. Geometrically, the dipoles had a spherical shape with a diameter of 100 nm, and electrically, they consisted of two poles of ±5 mV separated by an insulating layer of techotane material (V = 0 V). The electric potential was assigned on the two poles using Dirichlet boundary conditions. All the modelling of the MENP is consistent with Section 2.1 and corresponds to the maximum electrical polarization resulting on the MENP surface. The PPy nanofiber section in which MENPs were dispersed was modelled as a cylindric section with diameter of 500 nm, i.e., within the range reported in the literature (Xue et al., 2019), and with height equal to 15 μm (an assumption in the model resulting as a trade-off with computational costs). The electric conductivity of PPy was of 1000 S m^-1^, as in Section 2.1. Three different nanoparticle concentrations were considered: weight/volume (w/v) equal to 10%, 30% and 50%, corresponding to a total of 105, 316, and 526 MENPs, respectively. For each concentration, 50 random MENPs distributions were simulated.

Each polymeric section was then inserted within the nervous tissue, here approximated as a parallelepiped of 12 μm × 12 μm × 80 µm with dielectric properties of nerve tissue (Baumgartner et al., 2025) at the MENP stimulating frequency. To quantify the electric potential distribution (V) at the maximum electrical polarization of the MENPs, the electromagnetic ohmic quasi-static approximation and a finite element method were used to solve the Laplace equation:

$$\nabla \cdot \;\left(\sigma \;\cdot \nabla V\right)=0,$$ (1)

where V is the electric potential and σ (S m^-1^) is the electrical conductivity of tissues set at the MENP stimulation frequency. The electric field distribution *E* in all the modelled tissues was then obtained via the following relation (Equation 2):

E=−∇V. (2)

A rectilinear, uniform mesh with a 4.5 nm maximum step was used to discretize the cylindrical polymeric matrix, with fine grid increments up to 25 μm elsewhere, resulting in a total of approximately 66 Mcells. Dirichlet boundary condition with V = 0 was used to define the potential at the boundary of the computational domain. At this step, the simulations were implemented using the Sim4life platform (by ZMT Zurich Med Tech AG, Zurich, Switzerland, www.zurichmedtech.com).

To extract the electric potential distribution on the surface of each single unit, results were then exported into the MATLAB (R2023a) environment and post-processed.

### 2.3 Modelling of the poly-ME devices

The poly-ME devices were then modeled (Figure 1B), consisting of multiple nanofibers with dispersed MENPs of equal diameter and length, arranged in parallel and with fiber inter-distance hypothesized to be constant. To evaluate the effect of geometry of the device, two different geometries were modeled: a shorter and wider (short-wide) consisting of twelve nanofibers with a length of 150 µm (size: width × length × depth of 6.55 µm × 150 μm × 500 nm) and a longer and narrower (long-narrow) consisting of seven nanofibers with a length of 240 µm (size: width × length × depth of 3.80 µm × 240 μm × 500 nm). The area (width × length) of the two devices was comparable and resulted to be almost five times smaller than the surface of a TIME active site (Strauss et al., 2023). As a last investigation, to provide a first evaluation of the effect of device size, the surface area of the short-wide device was incremented fivefold, to approximate a TIME active site surface area. To define the electric potential distribution on the surface of the devices, the results of the electric potential distribution on the surfaces of every single unit were used. From the pool of post-processed electric potential distributions on the surface of single units at a constant MENPs concentration, distributions were randomly chosen and spatially rearranged to simulate the electric potential distribution on the surface of the devices with varying size and geometry. The electric potential distributions were then geometrically interpolated to the geometric models. All these steps were performed within the COMSOL Multiphysics software and the MATLAB (R2023a) environment.

### 2.4 Study of the nerve and poly-ME device interaction

The stimulating potential of the poly-ME devices was evaluated within a peripheral nerve model (Figure 1C), and a hybrid modeling approach, consisting of the solution of a volume conduction problem first and a neural response problem secondly (Romeni et al., 2020; Verardo et al., 2026), was used. The nerve was modelled using a simplified cylindrical geometry, with a length of 3 mm, a diameter of 2 mm and material properties of the perineurium (σ = 0.083 S m^-1^). At the centre of its cross-section, a cylindrical fascicle (σ = {0.083, 0.083, 0.571} S m^-1^) with a diameter of 240 μm was included. All sizes were based on morphological distributions of the human cervical vagus nerve, as in (Verardo et al., 2025). In addition, fibers were positioned in the cross-section of the fascicle and discretized in compartments along their longitudinal direction. This resulted in the definition of fiber nodes, i.e. points along the fibers where the extracellular potential is imposed (Romeni et al., 2020). The extraneural space was approximated by a cylindrical saline bath (σ = 2 S m^-1^) with a diameter of 3 mm and a length equal to that of the nerve.

The stimulating source, i.e., the poly-ME device, was assumed at the centre of the fascicle of fibers, and two different orientations were considered: the longitudinal orientation, in which the height of the poly-ME device was aligned to the longitudinal direction of fibers, and the transversal orientation, in which the height of the poly-ME device was oriented perpendicular to the longitudinal direction of fibers (Figure 1C).

The volume conduction problem, i.e., the quantification of the extracellular potential in the nerve tissue consequent to the electrical stimulation performed by poly-ME devices, was computed under the quasistatic approximation by solving Equation 1. COMSOL Multiphysics (COMSOL, 2024) software was used for the simulations.

### 2.5 Fibers distribution and neural computational simulation

The electrical stimulation of the poly-ME devices was evaluated by systematically quantifying the influence of two factors on the dynamic activity of the fibers: the distance of the fibers from the stimulating source, and the fiber type (i.e., myelinated and unmyelinated fibers).

All distances were defined as the transversal distance from the edge of the device to the fiber length (Figure 2). In the case of the poly-ME device oriented longitudinally, a total of six distances were considered, either with increased longitudinal or lateral distance from the device, in a L-shape distribution. By evaluating the influence of the longitudinal distance between the fiber and the device, the centre of the device was hypothesized aligned to the fiber length and located at increased distances of 0.5 μm, 1 μm, 5 μm and 10 μm, referred to as d1L, d2L, d3L and d4L, respectively. Vice versa, to evaluate the influence of the lateral distance, the centre of the device was kept at a longitudinal distance of 0.5 μm from the fiber length and moved laterally of 5 μm and 10 μm, referred to as d5L and d6L, respectively. In the case of the poly-ME device oriented transversally, a total of three distances were evaluated: 0.5 μm, 5 μm, and 10 μm, referred to as d1T, d2T, and d3T, respectively, by assuming the fibers centered at the device half height.

**FIGURE 2:**
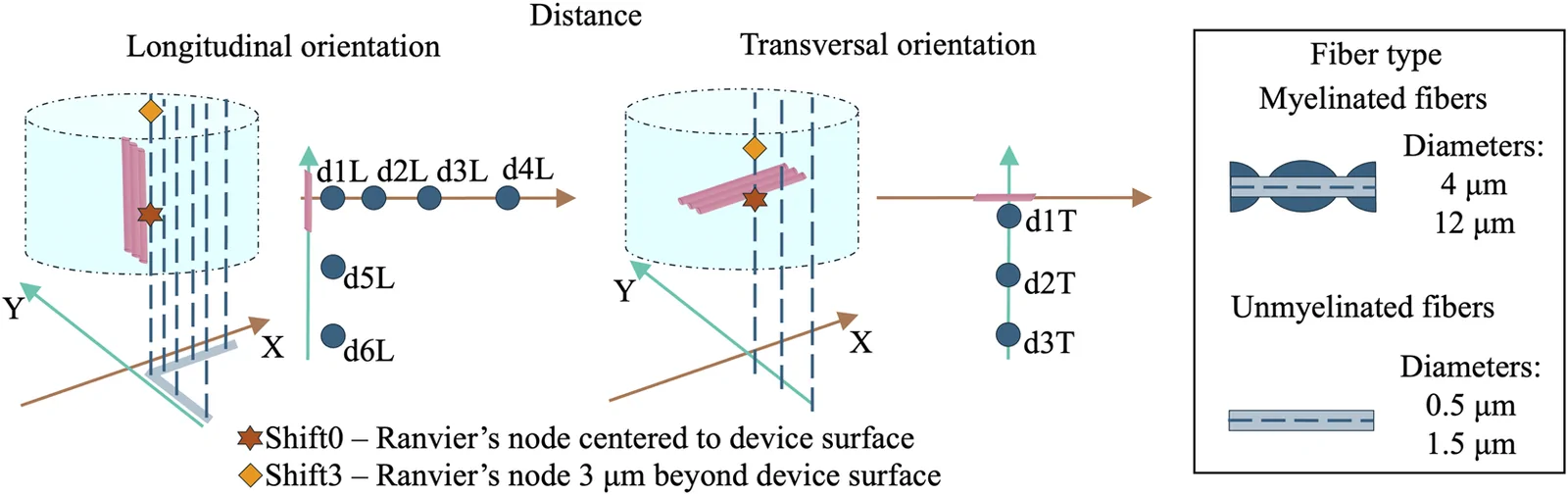
Left: Distances between the nerve fiber and stimulating source at longitudinal and transversal orientation of the stimulating device within the nerve fascicle (light blue). Right: Nerve fiber types considered.

At all distances, the activating function according to Rattay formalism (Rattay, 1998) was used to estimate the depolarization and hyperpolarization along the fiber length and the responses of both myelinated and unmyelinated fibers with varying fiber diameter were analyzed. Multicompartmental axon models were used to simulate the dynamic activity of fibers. The MRG model (McIntyre et al., 2002), which spatially discretizes the fiber in compartments representing the Ranvier nodes, paranodal segments (MYSA), juxtaparanodal segments (FLUT), and internodal segments (STIN), was used for myelinated fibers. Both small-myelinated and large-myelinated fibers were analyzed, with fiber diameters set at 4 μm and 12 μm, respectively. Under optimized conditions, it was hypothesized that a single Ranvier’s node of each fiber would be centered on the poly-ME device (shift0). Additionally, the influence of a longitudinal shift of the Ranvier’s node of 3 μm from the edge of the device was characterized (shift3). The Sundt (Sundt et al., 2015) model was used for the unmyelinated fibers, with a spatial discretization of 50/6 μm. Fiber diameters were set at 0.5 μm and 1.5 μm, representing Aβ, Aδ/B, and C fiber types from the Erlanger-Gasser classification (Gasser, 1941).

The temporal variation of membrane potential of nerve fibers was estimated by solving the neural response problem (Romeni et al., 2020, Verardo et al., 2026). At first, the extracellular potential imposed by the electrical stimulation of the device at the maximum electrical polarization of the MENPs resulting from the solution of the volume conduction problem was interpolated to fiber nodes. The distribution of fiber nodes was contingent on fiber dynamics and diameter, and the resulting sampling, corresponding to the trans-resistance calculated through the volume conduction problem, was referred to as lead-field matrix (LFM). Then, the solution of the LFM were scaled over time according to the stimulating waveform, here consisting of a charge-balanced biphasic stimulus with duration of 600 ms (Zhang et al., 2022). To identify the activation threshold, i.e., the point at which a spike occurred in the fiber, a binary search was conducted in which the amplitude of the stimulation waveform was increased. This method was employed to determine the necessary amplification of the electric stimulation of the devices required to achieve the activation of fibers, with the artificial amplification referred to as multiplication factor. The compartmental models and the neural stimulation were simulated with Neuron (Carnevale and Hines, 2006) through its Python interface.

### 2.6 Data analysis

The electric field on the surface of the polymeric sections and on the surface of the poly-ME devices obtained at the maximum electrical polarization of MENPs was quantified to evaluate the electrical stimulation exerted on the nerve tissue. Results were statistically analyzed to evaluate the effect of MENPs concentration, and the coefficients of variation served to evaluate the effect of random MENPs distribution within the polymer.

To assess the outcomes of the exerted electrical stimulation on the nerve tissue, results were evaluated in terms of extracellular electric potential along the fiber length and multiplication factors, as a proxy for the evaluation of the probability of eliciting a fiber response. The effects of MENP concentration, poly-ME device size and orientation, relative distance of the fibers from the stimulating device, and the influence of the neural dynamics were quantified.

## 3 Results

### 3.1 Electric field intensities generated by individual and random distributions of MENPs

From the characterization of the direct ME effect of a single MENP, Figure 3A shows the typical dipole-like behavior of the electric potential distribution resulting on the surface of the MENP at its maximum polarization. The maximum electric potential difference was equal to 10 mV, corresponding to a ME coefficient of 0.17 V cm^-1^ Oe^-1^. The electrical quantities on the surface of electrospun conductive PPy nanofiber sections that incorporate MENPs at varying concentration and random distribution were then characterized at the maximum MENPs polarization. Concentrations levels were equal to 10%, 30% and 50% weight/volume (w/v), and 50 random distributions for each concentration level were considered. Figures 3B,C show a qualitative representation of both the electric potential and electric field distributions on the surface of polymeric sections, respectively. As a direct consequence of the dipolar-like electric potential distribution on the MENPs’ surfaces and their relative orientation to the height of the polymeric sections, a more positive and a more negative electric potential distributions (Figure 3B) can be distinguished on the surface of the polymers, which are strongly affected by both nanoparticle concentration and distribution. The resulting electric field distributions (Figure 3C) were higher and more homogeneous on the surface of the nanofibers at the increase of MENP concentration, with variability on the shape of the distribution due to MENPs distribution within the polymeric matrix. To provide a quantification of the results, Figure 3D (top) shows the descriptive statistics (25th, 50th, and 75th percentiles) of the electric field distributions on the surface of each nanofiber section at varying MENPs concentration and distribution, overlapped with the corresponding averaged values at varying concentration. Results confirmed the increase in the electric field distribution at the increase in MENP concentrations. For example, the averaged 25th percentiles (dashed yellow lines) were equal to 1.6⋅10^3^ V m^-1^, 3.3⋅10^3^ V m^-1^ and 4.3⋅10^3^ V m^-1^ for w/v of 10%, 30% and 50%, respectively. At last, to quantify the variability of the results, Figure 3D (bottom) shows the coefficient of variation of the averaged 25th, 50th and 75th percentiles at varying MENPs concentration, which evidence a decrease in the variability at the increase of concentration. As an example, the coefficients of variation for the 25th percentiles are equal to 5.4, 3.2 and 2.8 for w/v of 10%, 30% and 50%, respectively. Taken together, results evidence how the increased concentration of nanoparticles results in a higher probability of them being distributed close to the surface of the nanowires and to each other, leading, in turn, to less variability in the electrical output across different random configurations.

**FIGURE 3:**
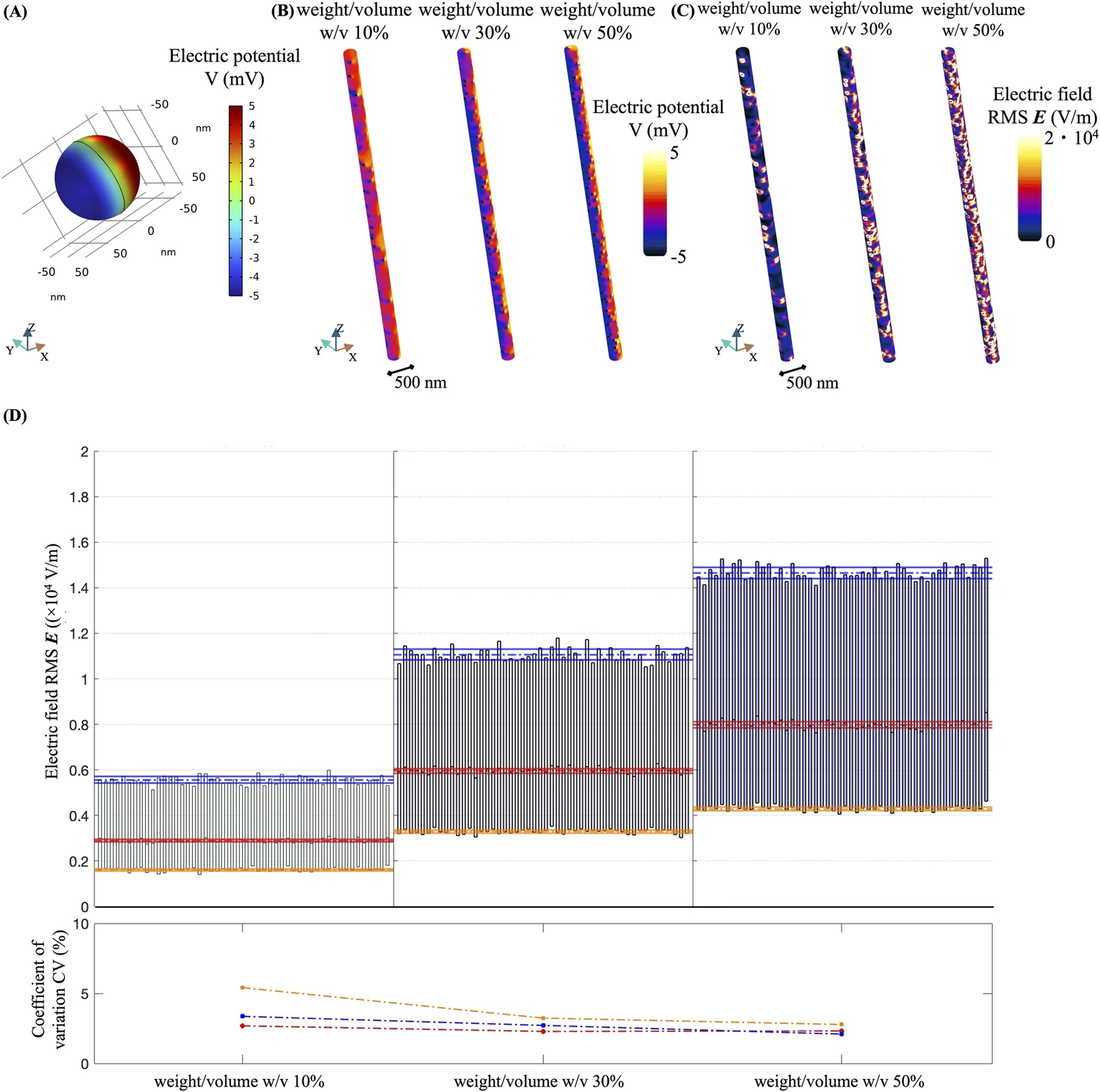
**(A)** Electric potential distribution on a surface of a single MENP; **(B,C)** Representative **(B)** electric potential distribution and **(C)** corresponding electric field distribution on the surface of conductive PPy nanofiber sections with random MENPs distribution and increasing concentration (weight/volume equal to 10%, 30% and 50%, respectively) are shown from left to right; **(D)** Top: statistical analysis of the electric field distribution on the surface of every of the simulated nanofiber sections with variable MENPs concentration and random distribution. The yellow, red, and blue lines represent the mean (dashed lines) and standard deviations (continuous lines) of the 25th, 50th, and 75th percentiles computed across all 50 random distributions at each constant w/v, respectively. Bottom: corresponding coefficient of variabilities.

### 3.2 Extracellular electric stimulation along nerve fibers’ length

Figures 4, 5 show the extracellular potential along the fiber length and its corresponding activating function when the short-wide poly-ME device with a surface area comparable to one-fifth of a standard TIME electrode’s active site is stimulating the nerve fascicle. Results are shown at varying orientation of the device (Figures 4, 5 correspond to the longitudinal and perpendicular orientations, respectively), at increasing MENP concentration (w/v of 10%, 30% and 50%) and at varying distance of the nerve fibers from the stimulating source, as shown in the legends of the figures. When the device has longitudinal orientation, nerve fibers experience a negative extracellular electric potential (Figure 4A) along their lengths, with peaks presenting varying width and amplitude and with higher variability in the extracellular potential profile seen at closer vicinity to the stimulating source. The corresponding activating functions (Figure 4B) show how the extracellular stimulation perceived by nerve fibers is highly inhomogeneous, alternating among depolarizing and hyperpolarizing stimulations with variable extension and intensity. When the device has transversal orientation, the extracellular potential profile and its activating function (Figure 5) dramatically vary. A positive and a negative electric potential peak are clearly seen, with the amplitude slowly decaying from the maximum value along the fiber length, and corresponding to a hyperpolarizing and a depolarizing stimulation, respectively (as shown by the activating function profile). As a general observation, irrespective of the device orientation, higher extracellular stimulation is achieved when the concentration of MENPs is increased and the distance of nerve fibers from the stimulating source is reduced. As shown in Figure 6, the most substantial increases in extracellular potential amplitudes occur when the concentration is increased from 10% to 30% w/v, rather than from 30% to 50% w/v. For example, when the device is longitudinally oriented and the nerve fiber is at distance d1L, the extracellular electric potential peak amplitude (in absolute value) along the fibers is 1.2 mV at 10% w/v, doubles to 2.4 mV at 30% w/v but reaches only 2.9 mV at 50% w/v. Vice versa, increasing the distance between the device and the fibers at a fixed MENPs concentration diminishes the amplitude of the extracellular electric potential along the fiber length. For example, when the device is oriented longitudinally at a nanoparticle concentration of 50%, the peak amplitude of the extracellular potential reduces from 2.9 mV at distance d1L to 0.7 mV at distance d6L. Correspondingly, in its transversal orientation, the peak-to-peak difference diminishes from 1.7 mV to 0.5 mV as the distance increases from d1T to d3T. As expected, the results show the lowest extracellular potential amplitudes at distances d4L, d5L and d6L when the device is longitudinally oriented, and the minimum peak-to-peak amplitude at distance d3T when the device is transversally oriented. Overall, given the rapid decay in amplitude of the electric potential in the extracellular space, the results suggest that the electrical stimulation of the poly-ME device remains primarily localized near its surface. Finally, from the analysis of the influence of the device geometry on the extracellular electric potential profile along the fiber length, results generally evidence negligible differences in amplitudes between the short-wide and the log-narrow devices (Figure 6), with consistent dependencies on the previously discussed variables (MENPs concentration, device orientation, and distance) across the two geometries. For example, at a weight/volume of 50%, longitudinal orientation and distance d1L, the extracellular electric potential peaks in absolute value were equal to 2.9 mV and 2.8 mV for the short-wide and the long-narrow geometries, respectively. Supplementary Figure S1 shows the extracellular electric potential profiles along the fiber length across the two geometries. The qualitative results across both orientations and all distances highlight the marginal difference in amplitude of the stimulation provided by the short-wide geometry compared to the long-narrow geometry, whereas differences in the extension of the stimulated fiber length are evidenced, with the long-narrow geometry eliciting a longer fiber’s length than the short-wide geometry.

**FIGURE 4:**
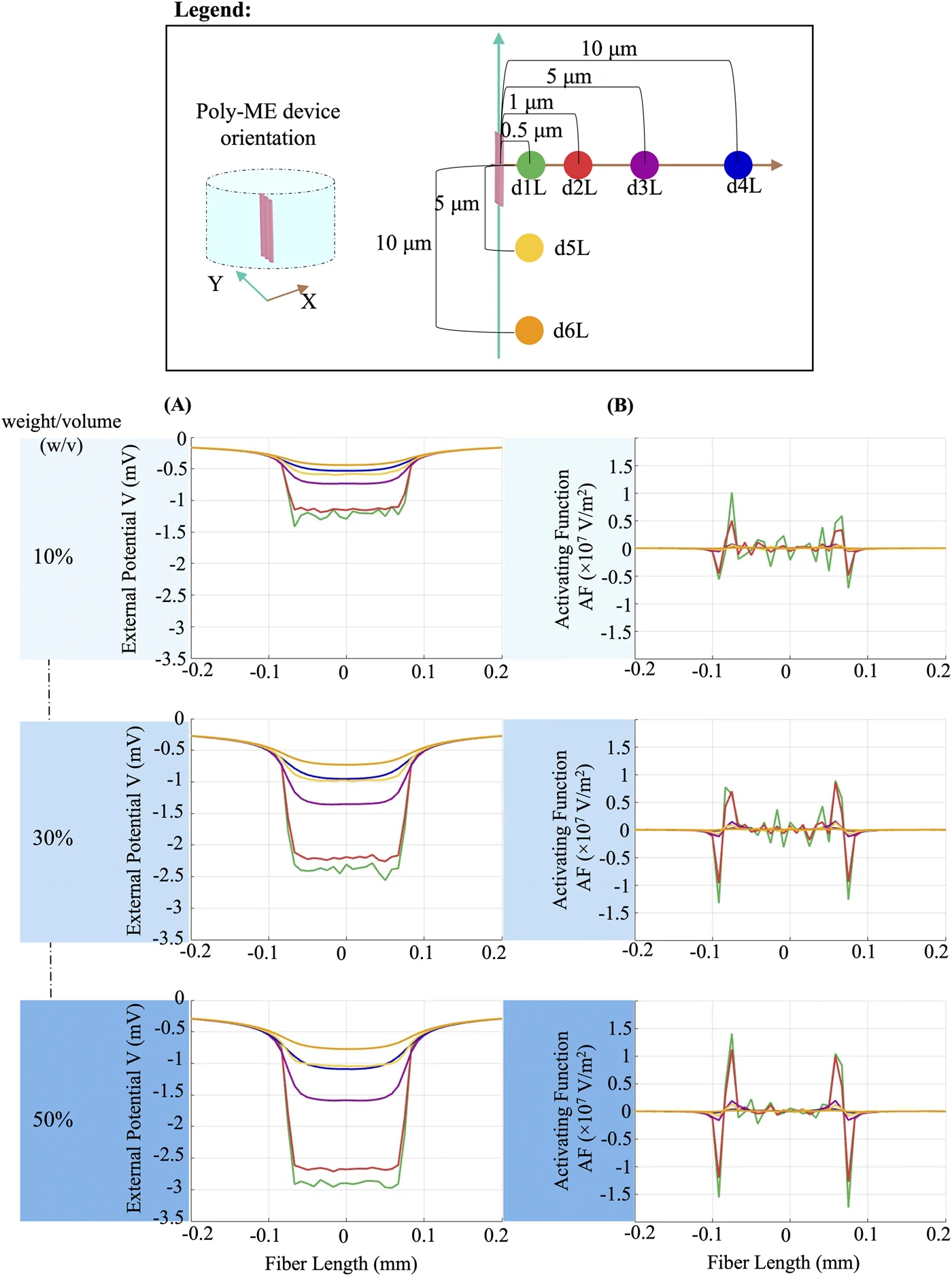
**(A)** Extracellular electric potential distribution along the fiber length and **(B)** corresponding activating function when the stimulating source is a short-wide small-size poly-ME device longitudinally oriented within a fascicle of fibers. Rows correspond to different MENP concentrations (10%, 30% and 50% weight/volume). The colors represent different distances between the nerve fiber and the stimulating source: d1L (green), d2L (red), d3L (purple), d4L (blue), d5L (yellow), d6L (orange).

**FIGURE 5:**
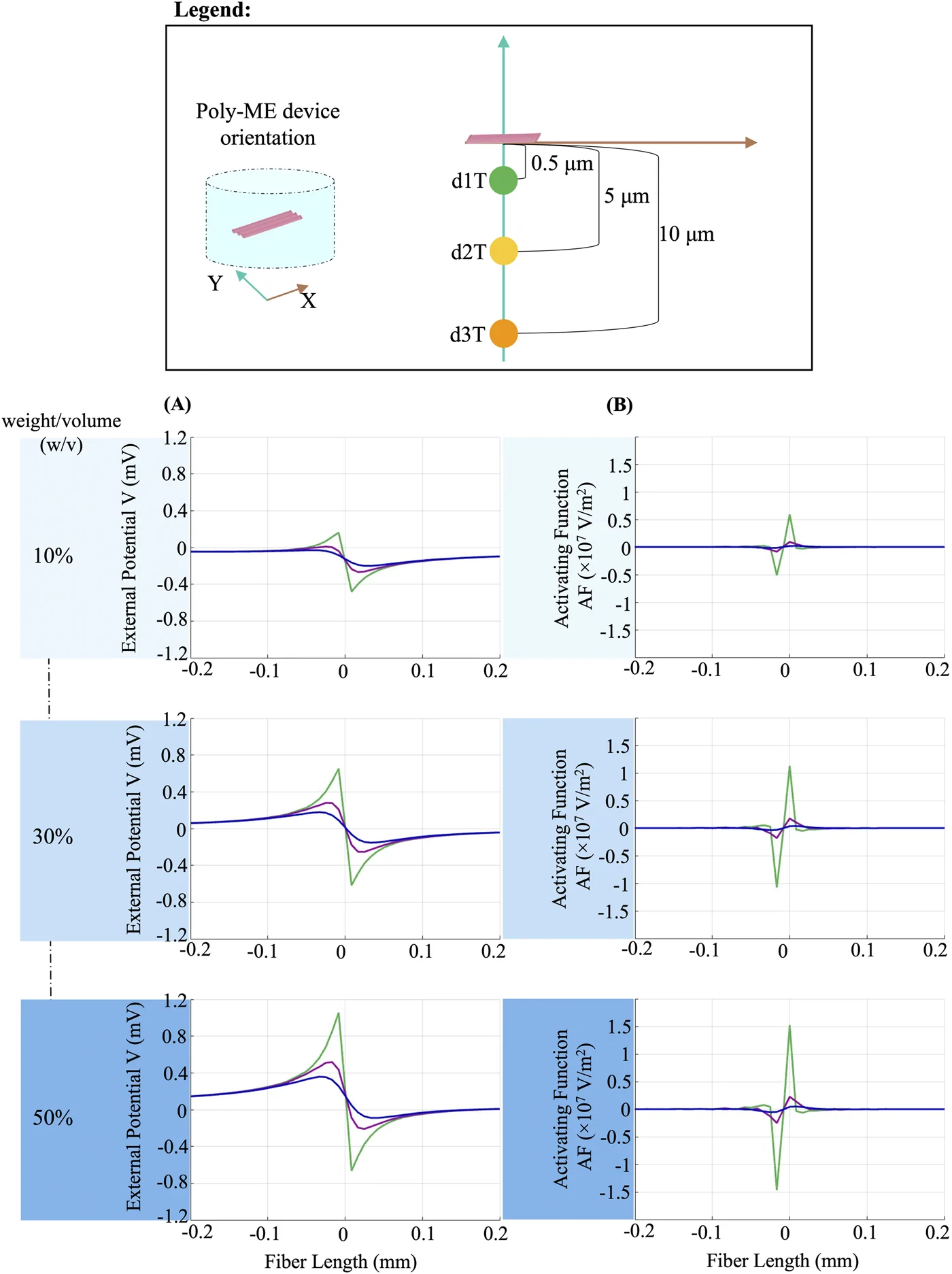
**(A)** Extracellular electric potential distribution along the fiber length and **(B)** corresponding activating function when the stimulating source is a short-wide small-size poly-ME device transversally oriented within a fascicle of fibers. Rows correspond to different MENP concentrations (10%, 30% and 50% weight/volume). The colors represent different distances between the nerve fiber and the stimulating source: d1T (green), d2T (yellow), d3T (orange).

**FIGURE 6:**
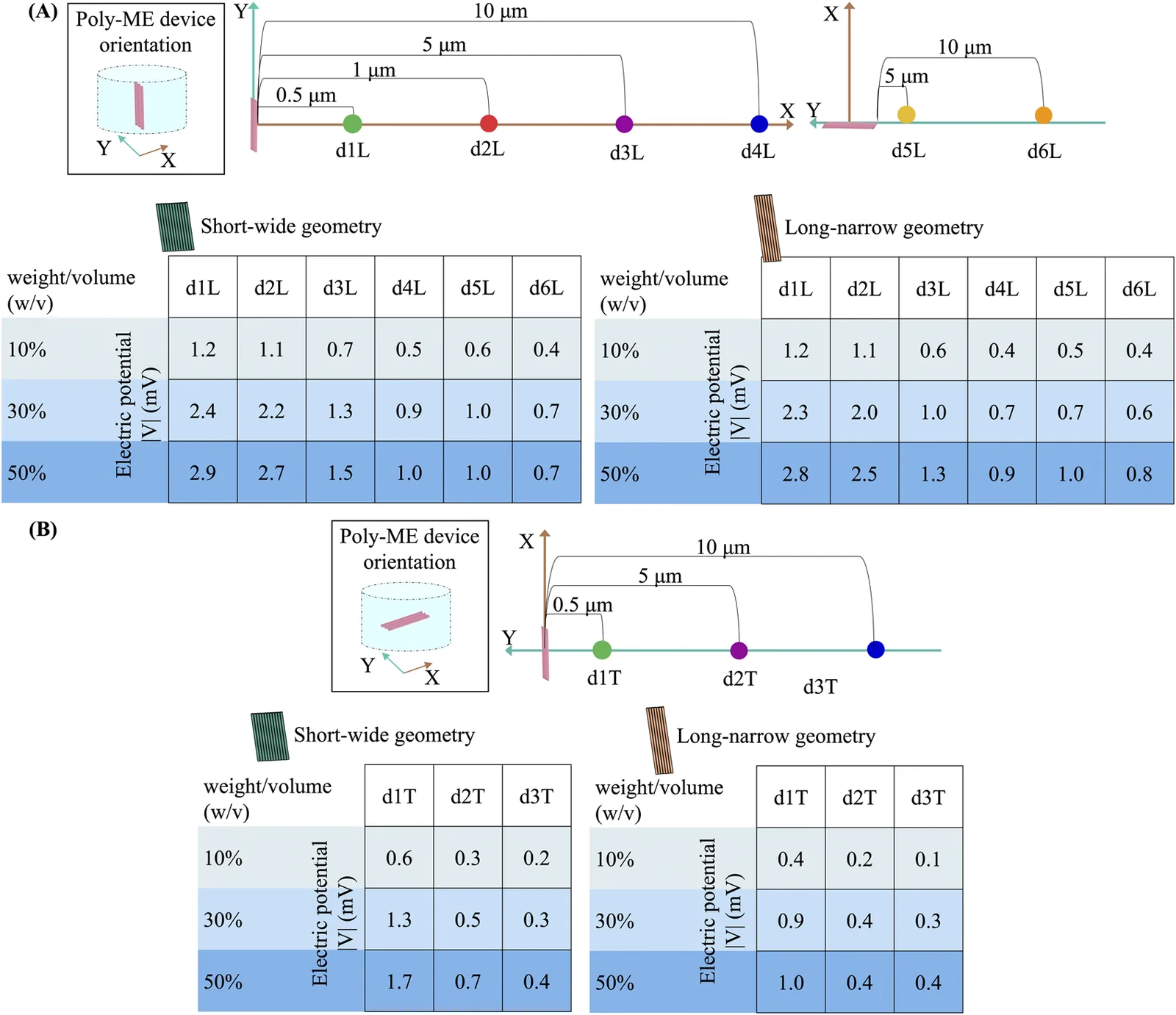
Influence of the poly-ME device geometry on the extracellular electric potential amplitudes perceived along the fiber length. **(A)** Absolute negative peak amplitude when the poly-ME devices are longitudinally oriented and **(B)** peak-to-peak difference when the poly-ME devices are transversally oriented. Results are shown at the three levels of MENP concentrations (rows) and different distances between the nerve fibers and the stimulating source (columns).

### 3.3 Characterization of the neuronal responses

Figure 7 shows the required multiplication factors, i.e., the artificial factors by which the exerted extracellular potential must be scaled to reach the activation threshold of a given fiber, to provide an indirect estimate of the recruitment of both myelinated and unmyelinated nerve fibers. Results are characterized at different device geometries and orientations, at varying MENPs concentrations, and at different distances of nerve fibers from the stimulating source for both myelinated and unmyelinated nerve fibers. When evaluating the effect of device geometry for each fiber type, similar multiplication factors are generally observed at longitudinal orientation and varying MENPs concentration, with the short-wide device typically requiring slightly lower amplification than the long-narrow one. For example, a multiplication factor equal to 19 is required for a 1.5 μm-diameter unmyelinated fiber at distance d1L when both geometries have a nanoparticle concentration of 50% w/v, but at distance d4L multiplication factors increase to 59 for the short-wide and 91 for the long-narrow geometry. In contrast, more pronounced differences in the multiplication factors between the two geometries are observed at transversal orientation of the device, but multiplication factors are mostly >100 for both geometries. The longitudinal orientation results in lower multiplication factors than the transversal orientation at comparable distances of the fiber from the stimulating source. For example, the multiplication factor for a 4 μm-diameter myelinated fiber located 5 μm from the stimulating source dramatically increases from the longitudinal (Galletta et al., 2025) to the transversal (>100) orientation at 10% w/v. Multiplication factors increase with decreasing MENPs concentration and increasing distance of the fiber, irrespective of device geometry and orientation and the fiber type. The lowest multiplication factors are observed at maximum MENPs concentration and minimum fiber distance. Specifically, at fixed distance, increasing MENPs concentration reduces multiplication factors. For example, the multiplication factor of a 12 μm-diameter myelinated fiber at distance d2L from a short-wide device longitudinally oriented decreases from 18 to 5 as concentration increases from 10% to 50% w/v. Conversely, at constant MENPs concentration, increasing distance raises the multiplication factors. For instance, for a long-narrow device longitudinally oriented at 30% w/v stimulating a 1.5 μm-diameter unmyelinated fiber, the multiplication factor increases from 27 to values > 100 at increasing the distance from d1L to d5L. Finally, fiber type strongly influences fiber recruitment. Myelinated fibers generally require lower multiplication factors than unmyelinated fibers when the device is oriented longitudinally, but a Ranvier node needs to be aligned with the device’s surface. Shifting the Ranvier’s node 3 μm beyond the device edge more than doubles the multiplication factors, reflecting the high sensitivity of myelinated fibers to their spatial alignment relative to the stimulating source. As the distance between the stimulating source and the node of Ranvier increases, the multiplication factors exceed that required for unmyelinated fibers–an effect most evident when the device is positioned transversally. The large variation in the results is primarily caused by the irregular extracellular potential at the nodes of Ranvier, which depends on their exact location relative to the stimulating device.

**FIGURE 7:**
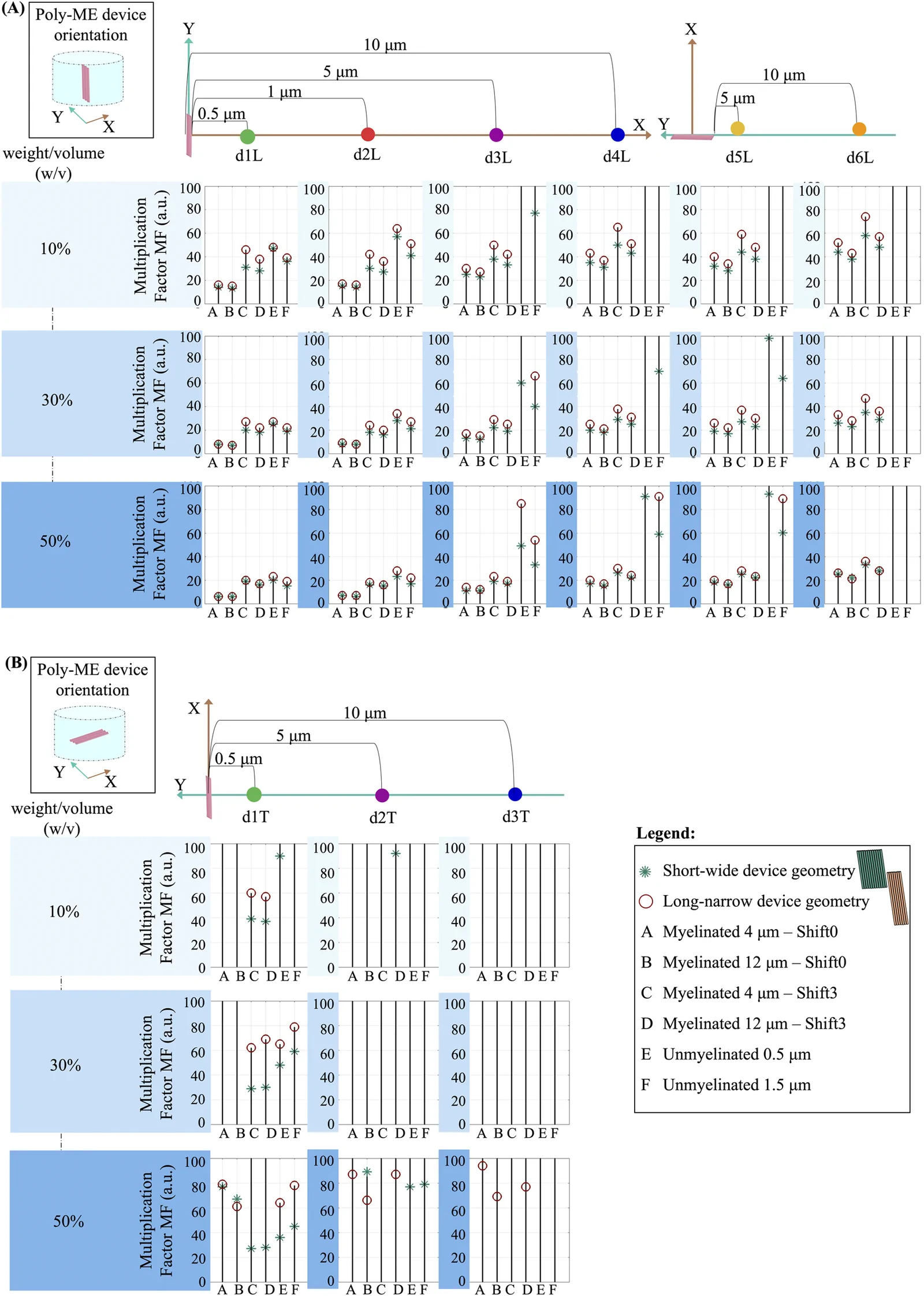
Multiplication factors required to elicit a neuronal response. Results for the short-wide (asterisk symbol) and long-narrow (circle symbol) poly-ME device geometries are compared. **(A)** Multiplication factors when the stimulating source is longitudinally oriented and **(B)** Multiplication factors when the stimulating source is transversally oriented. Columns correspond to evaluated locations: d1L, d2L, d3L, d4L, d5L and d6L for longitudinal orientation, and d1T, d2T and d3T for transversal orientation. Results are shown for the following fiber types: 4 μm-diameter myelinated with a Ranvier’s node centered on the stimulating device (A), 12 μm-diameter myelinated with a Ranvier’s node centered on the stimulating device (B), 4 μm-diameter myelinated with a Ranvier’s node shifted by 3 μm beyond the stimulating device (C), 12 μm-diameter myelinated with a Ranvier’s node shifted by 3 μm beyond the stimulating device (D), 0.5 μm-diameter unmyelinated (E) and 1.5 μm-diameter unmyelinated (F) fibers.

### 3.4 Quantification of the effect of the size of the poly-ME device

Figure 8 shows the multiplication factors resulting from the preliminary comparison evaluating how device size affects stimulating potential. Specifically, the large device had a surface area five times greater than that of the small device, thereby matching the surface area of a standard TIME electrode. Both devices had a short-wide geometry with 50% w/v and longitudinal orientation. The multiplication factors of the small device were reported previously in Figure 7. Overall, these results are consistent with the trends reported in Section 3.3. Lower multiplication factors are required for myelinated than unmyelinated fibers, but results are highly sensitive to the relative location of the Ranvier’s node to the stimulating source. In addition, irrespective of fiber type, increasing the distance of the fiber from the stimulating source resulted in higher multiplication factors. However, differences are seen across the two sizes. As expected, both sizes show similar multiplication factors at shorter distances (i.e., at d1L, d2L, and d3L), whereas at greater distances (i.e., at d4L, d5L, and d6L), the larger device outperforms the smaller one, with the multiplication factors more than halved. For example, a 4 μm-diameter myelinated fiber with a node of Ranvier centered on the stimulating source (node centered on the device, shift0) requires multiplication factors of 6 and 7 at distance d1L for both the small and large devices, respectively. At distance d4L, however, the multiplication factor drops to 9 for the larger device, compared to 17 for the smaller one.

**FIGURE 8:**
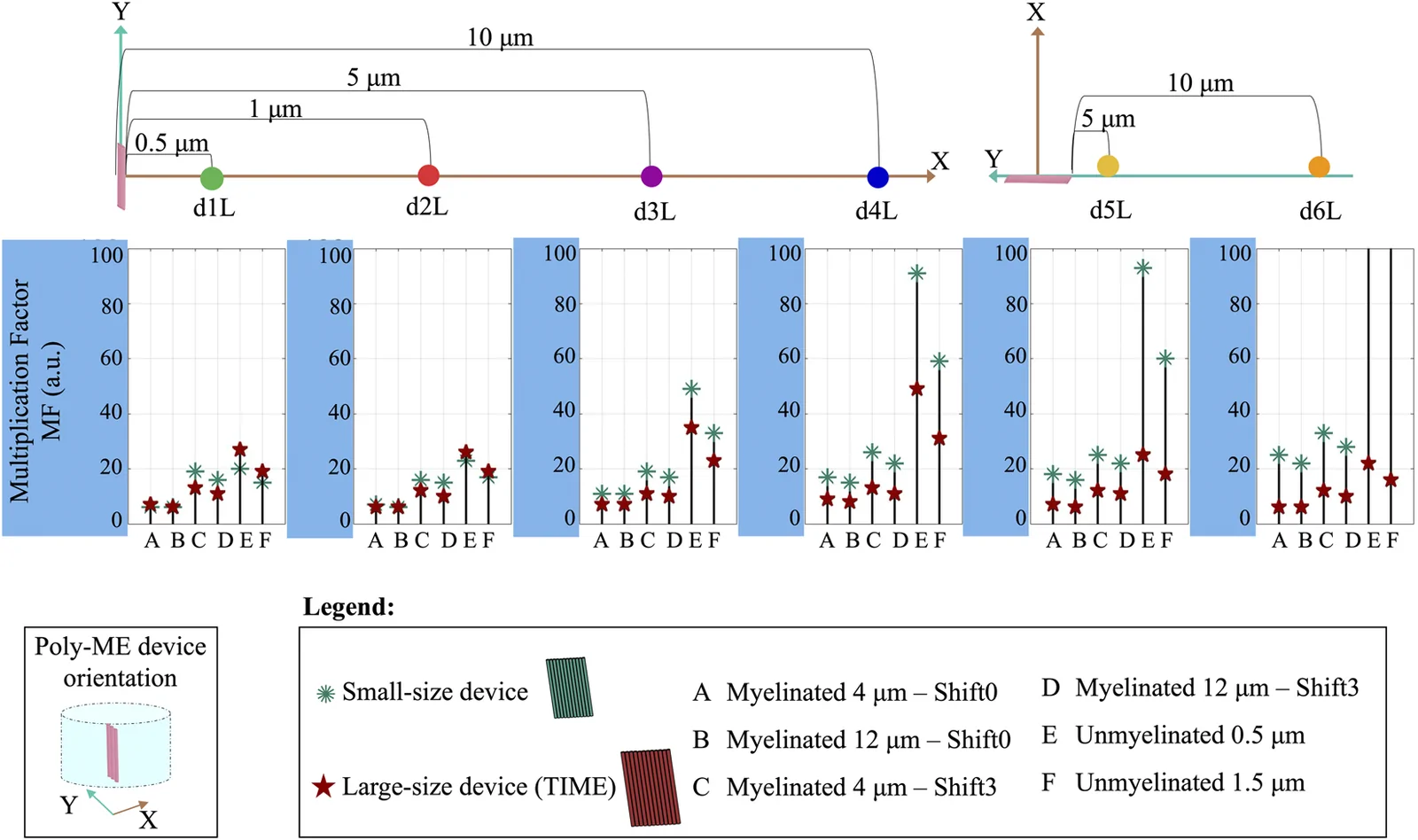
Multiplication factors required to elicit a neuronal response. Results for the small (asterisk symbol) and large (pentagram symbol) poly-ME device sizes are compared. The devices were simulated at a MENP concentration of 50% w/v and longitudinal orientation. Columns correspond to evaluated locations: d1L, d2L, d3L, d4L, d5L and d6L for longitudinal orientation, and d1T, d2T and d3T for transversal orientation. Results are shown for the following fiber types: 4 μm-diameter myelinated with a Ranvier’s node centered on the stimulating device (A), 12 μm-diameter myelinated with a Ranvier’s node centered on the stimulating device (B), 4 μm-diameter myelinated with a Ranvier’s node shifted by 3 μm beyond the stimulating device (C), 12 μm-diameter myelinated with a Ranvier’s node shifted by 3 μm beyond the stimulating device (D), 0.5 μm-diameter unmyelinated (E) and 1.5 μm-diameter unmyelinated (F) fibers.

## 4 Discussion

This study presented a comprehensive step-by-step characterization of the interaction between the innovative magnetoelectric nanoparticles and peripheral nerves by assuming the dispersion of MENPs within electrospun conductive polymer nanofibers. This approach enables a more homogeneous and controllable distribution of MENPs within the tissue while maintaining biocompatibility and compliance of the structure.

As the conductive polymer is the intermediary between MENPs (the electrical sources) and the peripheral nerve (the target tissue), its electrical, chemical, and mechanical properties strongly influence the combined properties of the MENPs–polymer complex. Electrically, a conductive polymer is essential for carrying charge from MENPs to the polymeric nanofiber surface in direct contact with the nerve tissue, enabling efficient delivery of electrical stimulation to the target tissue. In other words, a poorly conductive polymer would strongly attenuate the electrical output of MENPs, reducing in turn the stimulating effect within the target tissue, as quantified by (Suarato et al., 2025). Chemically, biocompatibility is mandatory for any material in direct contact with biological tissue to avoid any release of toxic substances. Conductive polymers have demonstrated high levels of biocompatibility (Grill et al., 2009; Navarro et al., 2005; Yildiz et al., 2020; Karagiorgis et al., 2025), and their use could preserve or further improve the demonstrated biocompatibility of MENPs. Mechanically, the compliance, flexibility, and biostability of the polymer strongly affect the mechanical match of the MENPs-polymer complex and its long-term functionality. A polymer that is flexible, compliant to the target tissue, and biostable minimize tissue disruption and remain chronically functional after implantation (Günter et al., 2019). Additionally, the encapsulation of MENPs within the polymer would also affect their clearance and excretion from the body–processes that are strongly influenced by the MENP properties (Apu et al., 2022) – and could extend their residence time in the targeted tissue. Given the wide range of electrical, chemical, and mechanical properties exhibited by conductive polymers (Karagiorgis et al., 2025; Xue et al., 2019), polymer selection must be guided by the specific requirements of the targeted applications. Based on previous successful applications (Hao and Yu, 2022; Zare et al., 2021; George et al., 2005), this study considers MENPs dispersed in conductive polypyrrole with conductivity of 1000 S m^-1^, which falls within the range of conductivities reported in the literature (Karagiorgis et al., 2025). Future studies should evaluate other polymers as candidate matrices for MENPs.

The possibility of dispersing MENPs in the polymer is translated into an indirect contact between nanoparticles and nerve tissue, therefore enabling the injection of higher concentration levels (here equal to 10%, 30%, and 50% w/v) than the concentrations typically injected directly into tissue (0.05%–5% w/v (Nguyen et al., 2021; Zhang et al., 2022; Kaushik et al., 2016; Guduru et al., 2015)). Yet, the dose-toxicity threshold for MENPs (Guduru et al., 2015) was satisfied. All chosen concentrations were within the range reported in the literature of nanoparticles dispersed in electrospun polymeric nanofibers (Xue et al., 2019; Asyraf et al., 2017). Consistent with previous work (Galletta et al., 2025), increasing MENP concentration has been demonstrated to yield an increase in electrical potential stimulation effect and a simultaneous reduction in the variability of the electrical quantities (Figure 3D) due to the higher probability of MENPs being distributed close together and closer to the polymer surface and the lower influence of their random distribution at higher MENP number (or concentration). The results show a greater increase in electrical stimulation between 10% and 30% w/v than the increase between 30% and 50%, which might suggest a saturation of the electrical effect of MENPs. However, it is important to emphasize that when the concentration is increased from 10% to 30% w/v, it is increased threefold. Therefore, to obtain a further increase with a similar result, we need to consider a concentration of 90%. Besides, lower concentrations are generally preferred to prevent MENP agglomeration, which would reduce the electrical stimulating effect of the MENPs, and to avoid obstructing the fabrication process of the MENPs and polymeric complex (Suarato et al., 2025). Taken together, these results suggest that the benefits of increasing MENP loading begin to decline after a certain point, therefore future studies should account for the trade-offs associated with MENP concentration.

The computational approach here used was a key tool to characterize the interaction between the MENPs dispersed in multi nanofibers polypyrrole structures (poly-ME devices) and peripheral nerve, with the probability of eliciting a neural response indirectly quantified in terms of multiplication factor. A lower multiplication factor corresponds to a higher feasibility of eliciting an action potential, with multiplication factors lower or equal than one indicating that the device output alone is sufficient to elicit the nerve fiber. Results evidenced the low influence of the geometry of the device and the great effect of the size of the device on its stimulating potential, with in general similar multiplication factors shown between the two considered geometries (Figure 7) and smaller multiplication factors shown for larger devices than smaller ones (Figure 8). However, increasing the surface area reduces spatial selectivity: a larger device stimulates a wider tissue volume, so higher stimulation strength comes at the loss of more precise targeting. In addition, the results confirmed the influence of the relative orientation of the stimulating source composed by MENPs on their stimulating potential, as previously evidenced in (Galletta et al., 2024). Given the typical electrical dipolar stimulation exerted on MENPs surfaces and the relative orientation of the MENPs to the nanofiber’s height, the device oriented longitudinally and the device oriented transversally exerted a monopolar-like or a dipolar-like electrical stimulation, respectively, and the results confirmed the higher efficacy of the longitudinal orientation which generally showed lower multiplication factors. When comparing multiplication factors across different myelinated and unmyelinated nerve fibers, the results (Figures 7, 8) confirm that non-linear neuronal dynamics influence the probability of activation of nerve fibers. Consistent with the literature (Lubba et al., 2019), myelinated fibers are more excitable than unmyelinated fibers. However, the excitability of myelinated fibers is significantly affected by the relative position of the node of Ranvier to the stimulating source. Lower multiplication factors are required for myelinated fibers compared to unmyelinated fibers when the device is oriented longitudinally, but this is true only under ideal conditions–when a node of Ranvier aligns with the stimulating source. As the node of Ranvier shifts away, the required multiplication factors increase. When the probability of a nodal alignment is further reduced, as for transversal orientation of the device, myelinated fibers show higher required multiplication factors and greater variability in the results than unmyelinated fibers. Overall, irrespective of nerve fiber type and across all scenarios of interaction between the stimulating poly-ME device and the targeted nerve fascicle evaluated, the multiplication factors exceeded one, evidencing the rare possibility of eliciting a nerve fiber response. However, even if the thresholds for nerve fiber activation can be far from being reached without amplification, subthreshold or near-threshold electrical stimuli can affect axonal excitability in peripheral nerves (Urriza et al., 2020), and then be converted into threshold stimuli through the integrative action of the axonal membrane, as in (Urriza et al., 2016; Naidoo and Jones, 2026). This opens a new scenario for the application of this technology to explore in future studies.

Compared to previous studies (Galletta et al., 2024; Galletta et al., 2025), this work dramatically advances the modeling of the MENPs-nerve electrical interaction through a step-by-step computational pipeline, allowing at the same time the evaluation of different factors of variability which are intrinsically inherent to this interaction. However, some assumptions and simplifications in the modeling are required. These aspects of the present work and its limitations are detailed in the following of this section. The model used to characterized the direct ME effect of MENPs leveraged of a previously validated methodology (Suarato et al., 2025; Marrella et al., 2023; Bonato et al., 2025; Fiocchi et al., 2022b), with more recent works employing it to provide an *in silico* quantitative prediction of the achievable local electric fields generated by MENPs at the particle scale for *in vitro* experiments (Cancino-Fuentes et al., 2026). The results of our study denoted a direct ME coefficient above magnetic saturation equal to 0.17 V cm^-1^ Oe^-1^, within the range of values reported in the literature (Kopyl et al., 2021; Wang et al., 2020). The first assumption was that all the nanoparticles randomly dispersed within the electrospun nanofibers were assumed to perform equally and in the absence of agglomeration. The second assumption was that the dipolar-like electrical potential distributions resulting on the MENPs surfaces were assumed to be perfectly aligned, i.e., under the hypothesis that the externally applied magnetic field used to activate the direct ME effect among the multiple randomly distributed MENPs was equal and that MENPs were perfectly aligned along their easy axis. Experimentally, these are ideal conditions which are difficult to achieve, where variability is intrinsic in the MENPs structure and can result in different activation capability. A first *in silico* characterization of the effect of variability can be found in a recent work (Bonato et al., 2026). Besides, several experimental approaches are currently being investigated to improve the electrospinning process of nanofibers containing dispersed MENPs and to promote nanoparticles alignment (Sreenivasulu et al., 2018; Cakmak, 2016). Further research is needed to fully characterize the phenomenon by addressing its various aspects of complexity, thereby overcoming its current limitations. The results also show that, despite the idealized conditions about the MENPs performance and orientation, the electrical stimulation generated by the poly-ME devices is insufficient to elicit an action potential. This finding limits the range of potential applications of such innovative devices and highlight the need to enhance their electrical output in future studies. A possible strategy would be to improve the performance of MENPs to exert a greater electrical stimulation in the target tissue. Because the direct ME coefficients span in a range from 0.001 V cm^-1^ Oe^-1^ to values greater than 1 V cm^-1^ Oe^-1^ (Fiocchi et al., 2022b), the performance of the MENPs considered in this study could be further increase to higher values. This result could be obtained by modifying the MENPs geometry, such its size and core–shell ratio, or by selecting different constituent materials–where the necessity of biocompatibility would be addressed by the interface of the polymeric structure between the MENPs and the biological tissue. Increasing the size of the ME sources at the micrometric scale, i.e., by assuming the use of thin-film magnetoelectric structures covered with a polymeric matrix, could provide an alternative solution to the use of MENPs, which could further increase electrical stimulation. Alternatively, another possible strategy to further increase the electrical output of MENPs would be to increase the frequency of the externally applied magnetic field used to elicit their direct ME effect to exploit their mechanical resonance. This approach could highly enhance the ME performance of MENPs (Caiani et al., 2026; Zaeimbashi et al., 2021), but its working frequency depends is related to the mechanical resonance, resulting in the GHz band for MENPs. This makes impossible to direct interact with the nervous system, requiring *ad hoc* rectifying solutions (Singer et al., 2020). A further limitation of the study is that, even if different configurations and variables are analyzed in detail, all the investigated scenarios included a single poly-ME within a single nerve fascicle. One potential solution would be to expand the computational workflow by including an automatic optimization protocol with machine learning approaches, similar to the approach outlined in (Romeni et al., 2023). This would facilitate the exploration of diverse architectural alternatives involving poly-ME devices, including the use of multiple poly-ME devices or the use of organic and inorganic structures in which to disperse MENPs. Additionally, optimizing the computational workflow could improve its effectiveness as a tool for providing quantitative, *in silico* predictions of the local electric fields generated by MENPs in experimental settings, as in (Cancino-Fuentes et al., 2026; Bonato et al., 2026). Besides, increasing the complexity of the stimulating structure would enlarge the area which is stimulated by the poly-ME devices. In the present work, due to the large sale difference between the spatial extend of the stimulation provided by the poly-ME devices and that of the nerve, simplified nerve geometry and nerve fibers paths were assumed in the model. However, both the natural tortuosity of the nerve and the heterogeneous distribution of nerve fiber diameters impact the stimulation generated by the source and its ability to recruit nerve fibers (Du et al., 2022; Du et al., 2021). Therefore, as the stimulated volume of the poly-ME devices is enlarged, future modeling should address this simplification by considering the morphological complexity of the nerve structure.

In summary, this work provides a computational framework for engineering MENPs-based stimulation of peripheral nerves and introduces electrospun conductive nanofibers as a MENPs delivery strategy. Embedding MENPs in a flexible polymer matrix allows higher MENP loading than direct tissue injection while improving surgical manageability, and the results of our study demonstrate the high localization of the electrical stimulation provided by these structures. The next step of this work will be experimental testing of the efficacy of using MENPs embedded in PPy for peripheral nerve stimulation. The findings presented here will provide useful guidelines for designing and optimizing the corresponding experiments, and our computational framework can serve as a versatile tool that can be adapted to different experimental setups to create a corresponding *in silico* model of the experimental test. To conclude, the synergy between the *in silico* model and the experimental design will be useful for accelerating the engineering of MENPs as stimulatory interfaces.

## Data Availability

The raw data supporting the conclusions of this article will be made available by the authors, without undue reservation.
