# Supplementary material for: Multi-scale computational modeling of magnetoelectric nanoparticle-dispersed conductive nanofibers for peripheral nerve modulation
Source: Front Bioeng Biotechnol. 2026 Oct 13;14:1951247. doi: 10.3389/fbioe.2026.1951247 (PMC13710225; doi:10.3389/fbioe.2026.1951247)
Supplement: Supplementary file 1 [file DataSheet1.pdf]

frontiers

Supplementary Material

Supplementary Table 1. Material properties of the MENP for multiphysics simulation.

	CFO core	***BTO shell***
Electrical conductivity	5.2×10^6 S/m	178.5 S/m
Density	5200 kg/m^3	5700 kg/m^3
Initial magnetic susceptibility	3	-
Saturation magnetostriction	-200 ppm	-
Saturation magnetization	2.47×10^5 A/m	-
Poisson's ratio	0.48	-
Young's modulus	230 GPa	-
Relative permittivity	10	{1115.1; 1115.1; 1251.3}
Elasticity matrix, voigt notation	-	{150.4; 65.6; 150.4; 65.9; 65.9; 145.5; 0; 0; 0; 43.9; 0;0;0;0;43.9;0;0;0;0; 0; 42.4} GPa
Coupling matrix, voig notation	-	{0; 0; -4.32; 0; 0; -4.32; 0; 0; 17.4; 0; 11.4; 0; 11.4; 0; 0; 0; 0; 0} C/m^2

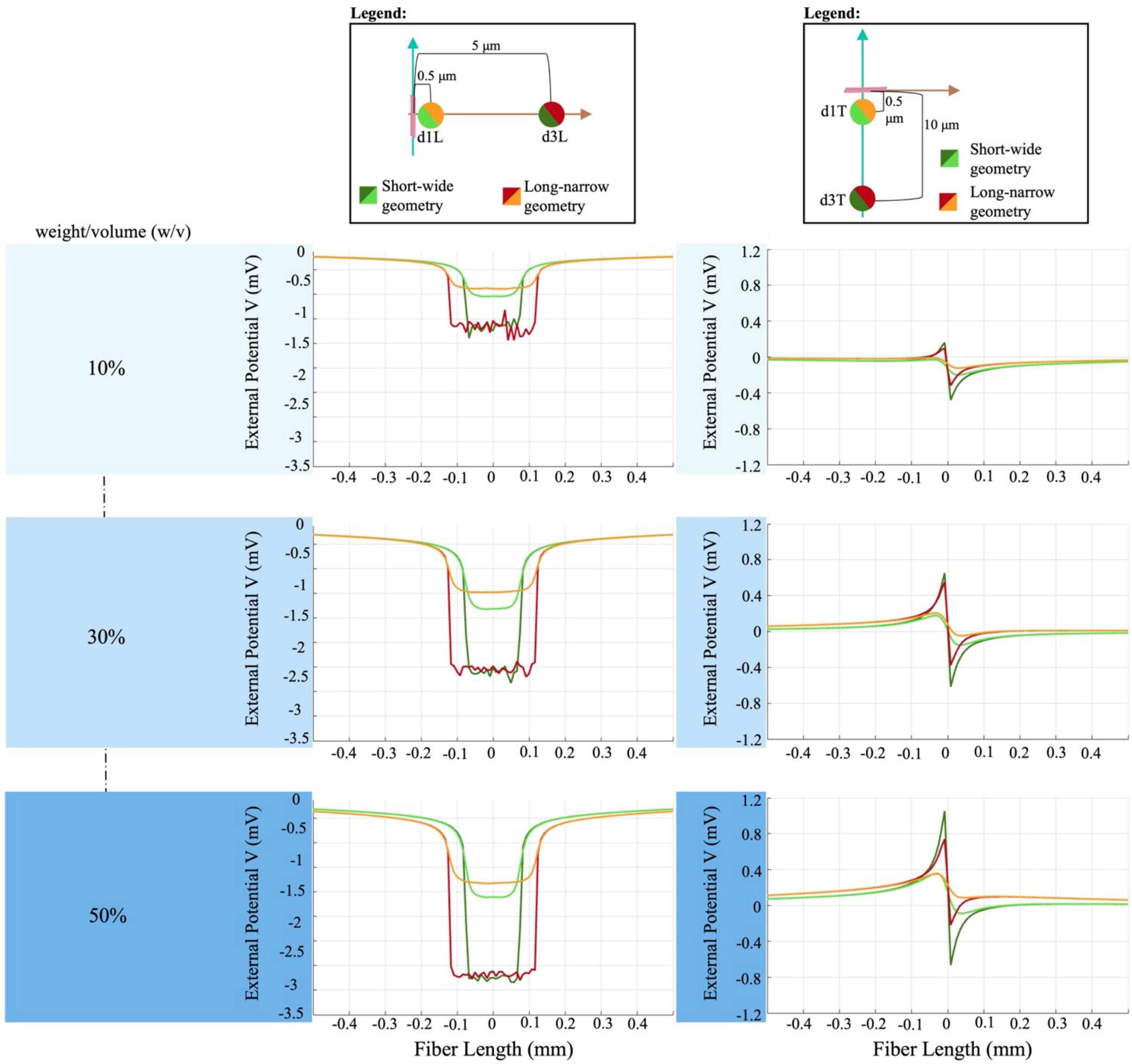

Supplementary Figure 1. Qualitative representation of the effect of device geometry on the extracellular electric potential distribution along the fiber length. Rows correspond to different MENPs concentration (10%, 30% and 50% weight/volume) and columns correspond to varying orientation of the poly-ME device within a fascicle of fibers (longitudinal on the left, and transversal on the right). The colors represent different distances between the peripheral fiber and the stimulating source and different device geometry.

COMSOL Multiphysics Model

In the following, details about the model of the single MENP described in Section 2.1 and implemented in COMSOL Multiphysics.

A 2D axisymmetric model was used to characterize the direct ME effect of a single MENP.

Geometry of the model:

- Circle 1: circle with radius of 45 nm centered in the origin of axis
- Circle 2: circle with radius of 50 nm centered in the origin of axis
- Square 1: square with size of 1 µm centered in the origin of axis
- Square 2: square with size of 1.025 µm centered in the origin of axis

From this geometric setting are obtained:

- Core (Circle 1)
- Shell (intersection of Circle 1 and Circle 2)
- Surrounding material (Square 1)
- Infinite Background (intersection of Square 1 and Square 2)

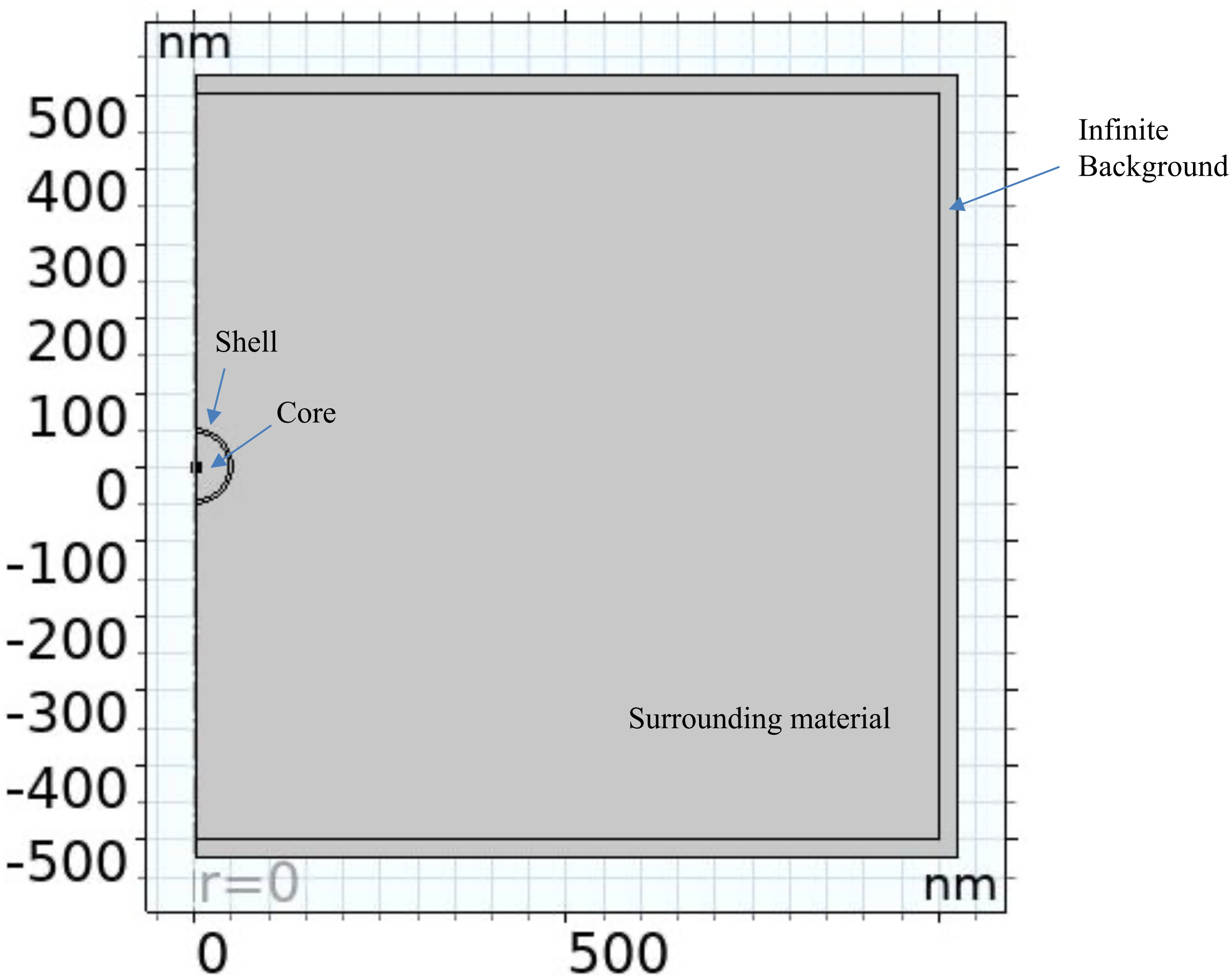

Boundary conditions:

The Infinite Background was defined as ‘Infinite Element Domain’ in ‘Artificial Domains’.

Physics Setup:

This step involved the use of three physics modules (*Magnetic Fields, Solid Mechanics, and Electrostatics*), which governing equations are described in the following of this section. The *Multiphysics* coupling was then used to couple the ‘Magnetostriction’ of the core and the ‘Piezoelectric effect’ of the shell (‘Coupling Type’ was ‘Fully Coupled’).
In all the modules, the ‘Axial Symmetry’ was defined

Magnetic Fields (mf) Module
The Ampère's law was solved:

$$\nabla \times \mathbf{H} = \mathbf{J} \quad (1)$$
$$\mathbf{B} = \nabla \times \mathbf{A} \quad (2)$$
$$\mathbf{J} = \sigma\mathbf{E} \quad (3)$$

with the constitutive relationship:

$$\mathbf{B} = \mu_0(\mathbf{H} + \mathbf{M}) \quad (4)$$

where **B** is the magnetic flux density, **A** is the magnetic vector potential, **J** is the current density, **E** is electric field and **M** the magnetization. **H** is the magnetic field value which is imposed on all the faces of Square 1 to simulate the application of the external magnetic field. Its amplitude along the z-direction is defined as ‘Hbias’ (previously defined in ‘Parameters’ in the ‘Global Definitions’ section).
The Jiles-Atherton model was used to described the magnetization of the cobalt ferrite core:

$$\mathbf{M} = \boldsymbol{M}_{rev} + \boldsymbol{M}_{irr} \quad (5)$$

where $\boldsymbol{M}_{rev}$ and $\boldsymbol{M}_{irr}$) are the reversible and irreversible components of the total magnetization, respectively.

Solid Mechanics (solid) Module
The equation of motion and strain-displacement relationship were used:

$$0 = \nabla \cdot \mathbf{S} + \boldsymbol{F}_v \quad (6)$$

$$\boldsymbol{\varepsilon} = \frac{1}{2}[(\nabla\boldsymbol{u})^T + \nabla\boldsymbol{u}] \quad (7)$$

where **S** is the stress tensor, $\boldsymbol{F}_v$ is the volume deformation tensor, $\boldsymbol{u}$ is the displacement vector, and $\boldsymbol{\varepsilon}$ is the strain tensor.
For the magnetostrictive core, the Hooke's law described the relationship between the stress and strain (which include its magnetostrictive strain). The magnetostrictive strain ($\boldsymbol{\varepsilon}_{me}$) for isotropic material is therefore expressed as:

$$\boldsymbol{\varepsilon}_{me} = \frac{3\lambda_s}{\mu_0 M_s^2} dev(\boldsymbol{M} \otimes \boldsymbol{M}) \quad (8)$$

where λ_s and M_s are the saturation magnetostriction and the saturation magnetization, respectively. For the piezoelectric shell, the stress-strain relationship includes both elastic and piezoelectric contributions:

$$\boldsymbol{S} = S_0 + \boldsymbol{C}: \boldsymbol{\varepsilon} + \boldsymbol{E} \cdot \boldsymbol{e} \qquad (9)$$

where $\boldsymbol{C}$ is the elastic right Cauchy deformation tensor, $\mathbf{E}$ is the electric field computed through the piezoelectric coupling and $\boldsymbol{e}$ is the piezoelectric Voigt coupling matrix representing the stress tensor.

Electrostatics (es) Module

The Gauss's law was solved to compute the electric potential distribution:

$$\nabla \cdot \mathbf{D} = \rho_v \qquad (10)$$

$$\mathbf{E} = -\nabla \mathrm{V} \qquad (11)$$

where $\mathbf{D}$ is the electric flux density, ρ_v is the volume charge density, and V is the electric potential. To couple the electric displacement and the mechanical strain in the piezoelectric shell, the piezoelectric constitutive equation was used:

$$\mathbf{D} = \varepsilon_0 \mathbf{E} + \varepsilon_0 \chi \mathbf{E} + \boldsymbol{e}: \boldsymbol{\varepsilon} \qquad (12)$$

where ε_0 is the vacuum electric permittivity and χ is the relative electrical susceptibility.

Mesh

‘Physics-controlled mesh’ with ‘Normal’ element size.

Computational Study

A ‘Parametric sweep’ and ‘Stationary’ study with ‘Physics controlled’ tolerance was solved. In the ‘Parametric sweep’ section, the ‘Hbias’ parameter was linearly varied in the range from 0 Oe to 10 kOe.
